# AHR is a tunable knob that controls HTLV-1 latency-reactivation switching

**DOI:** 10.1371/journal.ppat.1008664

**Published:** 2020-07-17

**Authors:** Weihao Hong, Wenzhao Cheng, Tingjin Zheng, Nan Jiang, Ruian Xu

**Affiliations:** 1 School of Medicine, Huaqiao University, Quanzhou, China; 2 Fujian Provincial Key Laboratory of Molecular Medicine & Xiamen Key Lab of Marine and Gene Drugs, Xiamen, China; 3 Engineering Research Center of Molecular Medicine, Ministry of Education, Xiamen, China; Penn State College of Medicine, UNITED STATES

## Abstract

Establishing latent infection but retaining the capability to reactivate in certain circumstance is an ingenious tactic for retroviruses to persist *in vivo* while evading host immune surveillance. Many evidences indicate that Human T-cell leukemia virus type 1 (HTLV-1) is not completely silent *in vivo*. However, signals that trigger HTLV-1 latency-reactivation switching remain poorly understood. Here, we show that aryl hydrocarbon receptor (AHR), a ligand-activated transcription factor, plays a critical role in HTLV-1 plus-strand expression. Importantly, HTLV-1 reactivation could be tunably manipulated by modulating the level of AHR ligands. Mechanistically, activated AHR binds to HTLV-1 LTR dioxin response element (DRE) site (CACGCATAT) and drives plus-strand transcription. On the other hand, persistent activation of nuclear factor kappa B (NF-κB) pathway constitutes one key prerequisite for AHR overexpression in HTLV-1 infected T-cells, setting the stage for the advent of AHR signaling. Our findings suggest that HTLV-1 might achieve its reactivation *in vivo* when encountering environmental, dietary, microbial and metabolic cues that induce sufficient AHR signaling.

## Author summary

HTLV-1 is considered largely latent *in vivo* because viral products were rarely detected in freshly isolated PBMCs of infected individuals. However, the existence of strong HTLV-1-specific immune response in most infected individuals suggests that the virus should not be completely silent *in vivo*. Since viral gene expression plays a critical role in cell transformation and *de novo* infection, a novel insight into where and how HTLV-1 achieves its reactivation *in vivo* is essential for developing new therapeutic approaches. AHR is a ligand-activated transcription factor that regulates intricate transcriptional programs in response to environmental, dietary, microbial and metabolic cues. It has been reported that AHR is constitutively overexpressed in HTLV-1-infected T-cells. Nevertheless, the functional role of AHR in HTLV-1 pathogenesis is still obscure. In this study, we show that activated AHR can directly bind to HTLV-1 LTR DRE site (CACGCATAT) and drive HTLV-1 plus-strand transcription. Importantly, HTLV-1 latency-reactivation-latency switching could be manipulated in MT-1 cells by adding and removing additional kynurenine (a well-known AHR ligand). Moreover, we explicate that the persistent NF-κB activation is critical for AHR overexpression in HTLV-1-infected T-cells. These results imply that constitutive AHR overexpression in infected T-cells endues HTLV-1 the potential to reactivate from latency when the level of AHR ligands reaches a certain threshold. Accordingly, we propose that HTLV-1 might achieve its reactivation in certain parts of the body that are prone to accumulate AHR ligands.

## Introduction

Human T-cell leukemia virus type 1 (HTLV-1) is a delta-type retrovirus that etiologically associates with adult T-cell leukemia (ATL) and several inflammatory diseases, such as HTLV-1-associated myelopathy/tropical spastic paraparesis (HAM/TSP) [1]. Following infection, HTLV-1 provirus integrates into the host cell chromatin and the long terminal repeats (LTRs) located at the 5’ and 3’ ends of the provirus act as promoters responsible for plus- and minus-strand transcription respectively. Most HTLV-1 genes are encoded in plus strand including *gag*, *pro*, *pol*, *env*, *tax*, *rex*, *p12*, *p13* and *p30*, except for the minus-strand gene, HTLV-1 bZIP factor (*HBZ*). Unlike HTLV-1-transformed T-cell lines expressing abundant amounts of viral products *in vitro*, HTLV-1 is considered largely latent *in vivo* because virions and viral proteins were rarely detected in freshly isolated peripheral blood mononuclear cells (PBMCs) of infected individuals. However, the presence of high titles of HTLV-1-specific cytotoxic T-lymphocytes (CTLs) and antibodies in most infected individuals suggests that the immune system is frequently stimulated by newly synthesized HTLV-1 antigens [2, 3]. Thus, HTLV-1 should not be completely silent *in vivo* [4, 5]. Given the critical role of viral gene expression in cell transformation and *de novo* infection, a better understanding of where and how HTLV-1 achieves its reactivation can lead to important insights for developing strategies to prevent and treat HTLV-1-associated diseases.

Aryl hydrocarbon receptor (AHR) is a member of the basic helix–loop–helix–PER–ARNT–SIM (bHLH-PAS) transcription factor family, which is originally discovered as the receptor that binds environmental pollutant 2,3,7,8-tetrachlorodibenzo-p-dioxin (TCDD, also known as dioxin) [6]. To date, a wide variety of ligands are known to activate AHR, including xenobiotic substances, dietary components, hememetabolites and tryptophan metabolites [7–10]. Activated AHR translocates from the cytoplasm into nucleus and heterodimerizes with AHR nuclear translocator (ARNT). The AHR-ARNT complex further binds to dioxin response element (DRE) located on the promoter of target genes, such as cytochrome P450 (CYP1) family members (*CYP1A1*, *CYP1B1*, etc.), and regulates their transcription. Emerging evidences suggest that AHR is not only an environmental sensor but also an assistor for many viruses to achieve their survival advantage. For instance, AHR signaling facilitates the replication of vesicular stomatitis virus (VSV), influenza virus (FluV), Sendai virus (SeV), encephalomyocarditis virus (EMCV) and herpes simplex virus type 1 (HSV-1) in mouse embryonic fibroblasts by elevating the expression of TCDD-inducible poly(ADP-ribose)polymerase (TIPARP), which in turn suppresses type I interferon-mediated antiviral defense [11]; Hepatitis C virus (HCV) upregulates AHR-CYP1A1 pathway to accumulate enlarged lipid droplets, thereby promoting viral assembly [12]; activated AHR binds to human immunodeficiency virus type 1 (HIV-1) LTR, leading to viral reactivation and enhanced viral infection [13–15]. In the context of HTLV-1 infection, it has been reported that AHR is constitutively overexpressed in HTLV-1-infected T-cell lines as well as primary ATL cells [16]. However, the functional role of AHR in HTLV-1 pathogenesis has never been explicated.

In this study, we show that ligand-activated AHR can directly bind to HTLV-1 LTR DRE site (CACGCATAT) and drive plus-strand transcription. Importantly, we found that HTLV-1 latency-reactivation-latency switching was controllable in MT-1 cells by adding and removing additional kynurenine (a well-known AHR ligand). These results suggest that HTLV-1 possesses the capability to reactivate from latency when the level of AHR ligands reaches a certain threshold. Thus, we reveal a previously unidentified mechanism which might shed light on where and how HTLV-1 achieves its reactivation *in vivo*.

## Results

### AHR signaling contributes to HTLV-1 plus-strand expression

**Fig 1:**
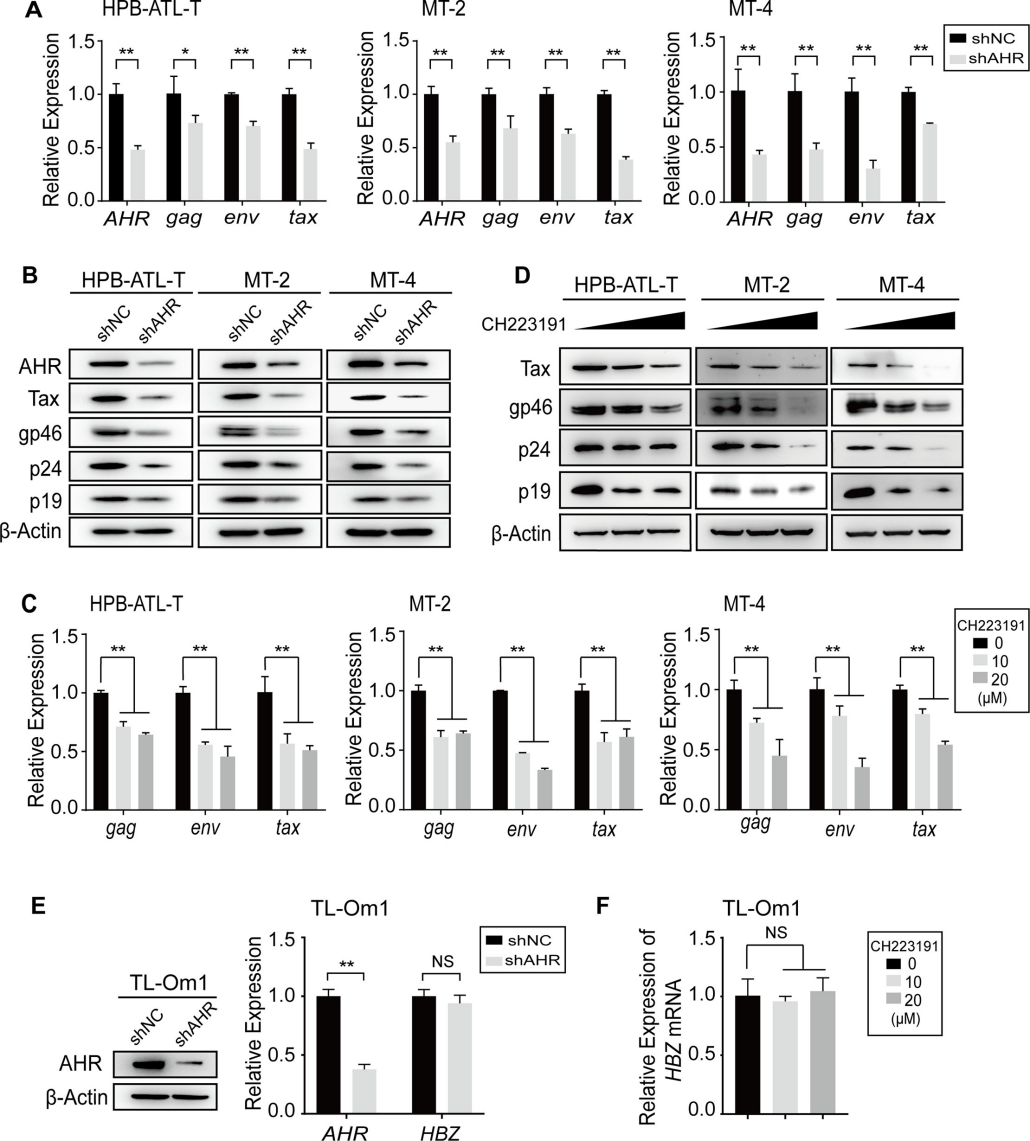
AHR signaling contributes to HTLV-1 plus-strand expression. (**A, B, E**) HPB-ATL-T, MT-2, MT-4 and TL-Om1 cells were infected with lentivirus against AHR. The screened AHR-knockdown cells were subjected to analyze the expression by (**A, E**) quantitative RT-PCR and (**B, E**) immunoblot. (**C, D, F**) HPB-ATL-T, MT-2, MT-4 and TL-Om1 cells were treated with CH-223191 (10, 20 μM) or DMSO for 24 h, then subjected to analyze the expression by (**C, F**) quantitative RT-PCR and (**D**) immunoblot. Results of quantitative RT-PCR are shown as relative mRNA expression normalized to that of 18S rRNA. shNC, negative control of shRNA. *, p<0.05. **, p<0.01. NS, not significant.

Due to the existence of endogenous AHR ligands (e.g. tryptophan metabolites) in cell culture medium [17, 18], background AHR signaling is ongoing in HTLV-1-infected T-cell lines without adding exogenous ligands. Since AHR signaling has been reported to be implicated in HIV-1 gene expression, an important question arises: would this background AHR signaling be associated with HTLV-1 gene expression? To investigate this possibility, we suppressed AHR expression in HPB-ATL-T, MT-2 and MT-4 cells using lentivirus-mediated short hairpin RNA (shRNA)—these three cell lines express abundant amounts of viral mRNA and proteins, thus they are suitable for detecting changes in the level of viral gene expression. We found that knockdown of AHR significantly reduced the expression of plus-strand genes, including structural gene *gag* (p24 and p19 matrix), *env* (gp46) and regulatory gene *tax*, at both mRNA and protein levels (Fig 1A and 1B).To further validate these observations, we used an AHR-specific antagonist CH-223191, which inhibits AHR nuclear translocation (S1 Fig). Consistently, compared with the control groups, treatment with CH-223191 greatly impaired plus-strand expression in HPB-ATL-T, MT-2 and MT-4 cells (Fig 1C and 1D).

Transcription of minus-strand gene *HBZ* is governed by a TATA-less, Sp1-predominant promoter in 3’LTR [19], and HBZ expression is generally inversely correlated with plus-strand expression, as HBZ impedes Tax-mediated transactivation of plus strand via sequestrating cyclic AMP-responsive element-binding protein (CREB) and the transcriptional coactivators CREB binding protein (CBP)/p300 [20, 21]. Hence, it is of interest to test if AHR signaling impacts HBZ expression. Given that Tax can also drive minus-strand transcription [19], we utilized a special ATL cell line, TL-Om1, in which plus-strand genes are silenced owing to the hypermethylation of 5’ LTR [22]. We found that both knockdown of AHR and treatment with CH-223191 did not affect *HBZ* transcription in TL-Om1 cells.

### Manipulation of HTLV-1 latency-reactivation-latency switching in MT-1 cells via adding and removing additional AHR ligands

**Fig 2:**
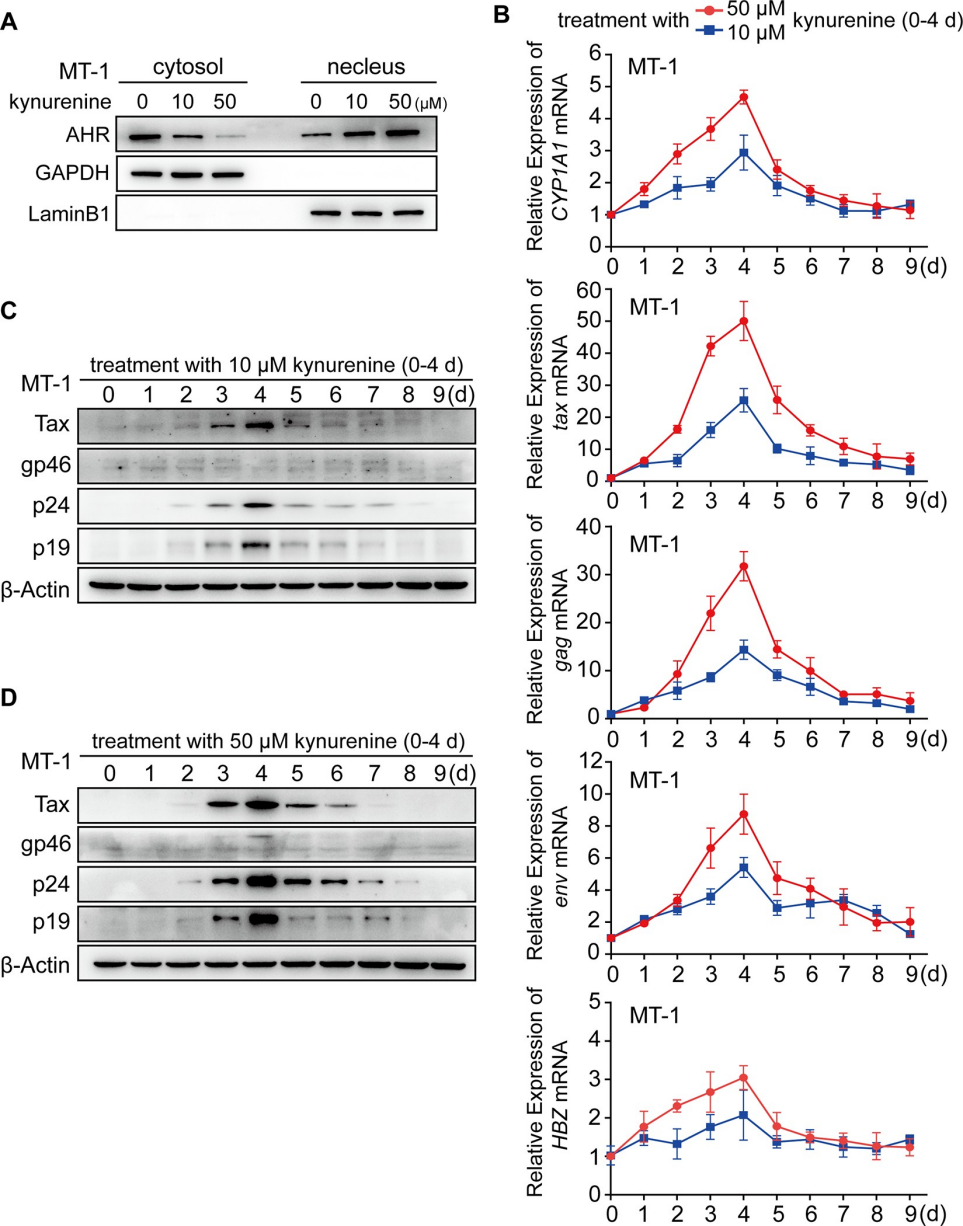
Manipulation of HTLV-1 latency-reactivation-latency switching in MT-1 cells via adding and removing additional AHR ligands. (**A**) MT-1 cells were treated with kynurenine (10, 50 μM) or DMSO for 48h, then subjected to analyze the level of cytosolic AHR and nuclear AHR by immunoblot. GAPDH and LaminB1 were used as control for cytosolic and nuclear fraction respectively. (**B**-**D**) MT-1 cells were treated with kynurenine (10, 50 μM) for 4 days then cultured in basic cell culture medium for another 5 days (culture medium was replaced every day). The expression was analyzed by (**B**) quantitative RT-PCR and by (**C**, **D**) immunoblot.

Unlike HPB-ATL-T, MT-2 and MT-4, some other HTLV-1-infected T-cell lines—e.g. MT-1, which is thought to have a pattern of viral gene expression equivalent to primary HTLV-1-infected T-cells [23, 24]—only exhibit faint expression of plus-strand genes under the background AHR signaling (when cultured in basic cell culture medium). We therefore tested whether additional AHR signaling is able to reactivate the latent virus in MT-1 cells. A well-known AHR ligand was used—the tryptophan metabolite, kynurenine [7] (Fig 2A). After 4-days treatment, kynurenine (10, 50 μM) remarkably induced the expression of *CYP1A1* (AHR target gene) and plus-strand genes (*gag*, *env* and *tax*) in a dose-dependent manner (Fig 2B). Both groups efficiently triggered the production of Tax protein, p19 and p24, although the accumulation of gp46 was no apparent (Fig 2C and 2D). Since Tax drives minus-strand transcription, it is not surprising that *HBZ* mRNA also increased slightly.

As Tax potently transactivates HTLV-1 LTR, there is no doubt that Tax should contribute to AHR-mediated HTLV-1 reactivation. The question was that, could the induced Tax sustain this reactivation without the additional AHR signaling? To address this issue, we incubated the cells in basic cell culture medium for another 5 days after the 4-days ligand treatment. Unexpectedly, the induced plus-strand expression gradually dropped after removal of the additional ligands, and Tax protein could not be detected in both groups on day 9 (Fig 2C and 2D), indicating that HTLV-1 latency was reestablished.

Taken together, these results suggest that (i) HTLV-1 possesses the capability to reactivate from latency when the level of AHR ligands reaches a certain threshold and (ii) AHR signaling is critical not only for initiating HTLV-1 reactivation but also for sustaining.

### Activated AHR binds to HTLV-1 LTR and drives HTLV-1 plus-strand transcription

**Fig 3:**
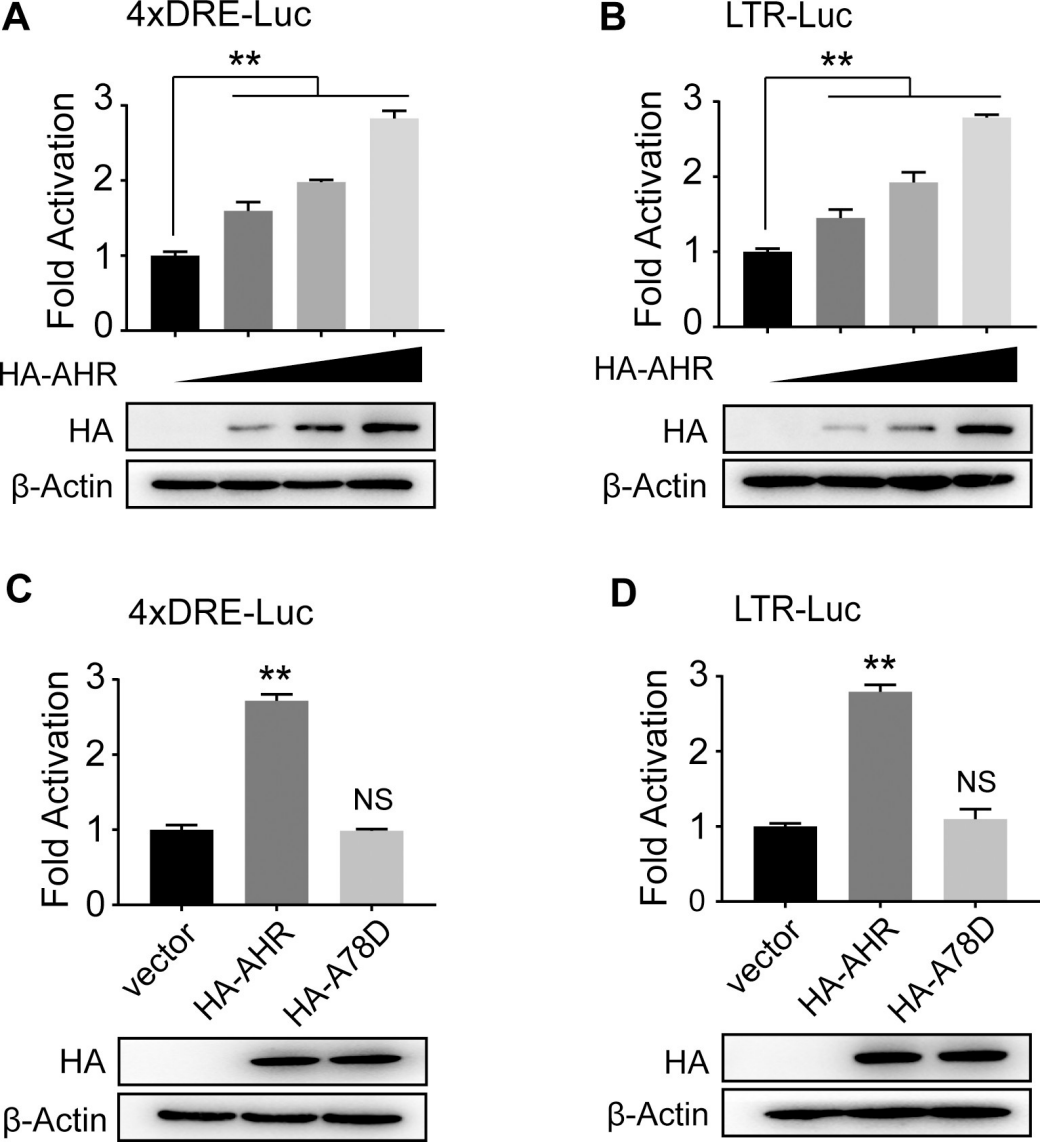
AHR transactivates HTLV-1 LTR in a DRE-dependent manner. (**A, B**) Dual luciferase reporter analysis in HEK293 cells co-transfected with (**A**) 4xDRE-Luc (0.5 μg) or (**B**) HTLV-1 LTR-Luc (0.5 μg) and increased pcDNA3.1-HA-AHR (0, 0.1, 0.2, 0.5 μg). (**C, D**) Dual luciferase reporter analysis in HEK293 cells co-transfected with (**C**) 4xDRE-Luc (0.5 μg) or (**D**) HTLV-1 LTR-Luc (0.5 μg) and pcDNA3.1/pcDNA3.1-HA-AHR/pcDNA3.1-HA-AHR^A78D^ (0.5 μg). For each sample, 20 ng pRL-TK was used as an internal control. Results are shown as relative luciferase activity (firefly/renilla) normalized to that of the control group. Expression of transfection was analyzed by immunoblot. **, p<0.01. NS, not significant.

Then, we investigated at the molecular level how AHR modulates HTLV-1 plus-strand expression. A reporter plasmid, 4xDRE-Luc [25], was employed as an indicator of AHR activation. We found that transfected AHR could activate 4xDRE-Luc without adding exogenous ligands (Fig 3A). This observation confirms the existence of endogenous AHR ligands in cell culture medium. Therefore, we tested whether transfected AHR transactivates HTLV-1 LTR. As shown in Fig 3B, transfected AHR activated HTLV-1 LTR-Luc in a dose-dependent manner. It has been reported that AHR can control gene expression through non-DRE response elements [26–28]. To determine if AHR-mediated transactivation of HTLV-1 LTR is DRE-dependent, we utilized an AHR mutant, AHR^A78D^, which lacks DRE-dependent transcriptional capability [29] (Fig 3C). We found that AHR^A78D^ failed to activate HTLV-1 LTR-Luc (Fig 3D), suggesting that AHR drives HTLV-1 plus-strand transcription in a DRE-dependent manner.

**Fig 4:**
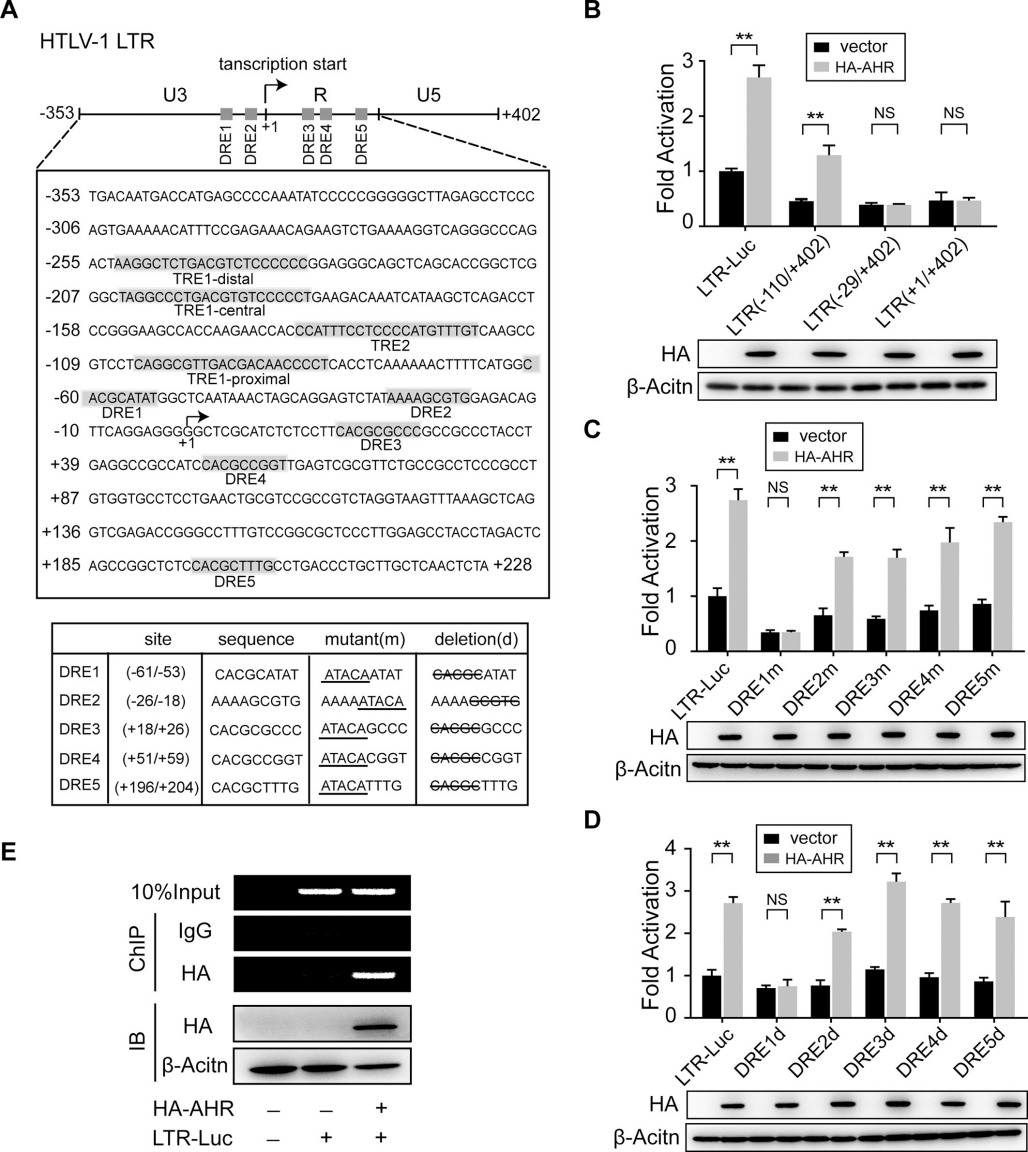
Activated AHR binds to HTLV-1 LTR and drives HTLV-1 plus-strand transcription. (**A**) Sequence map for HTLV-1 LTR U3 and R region (upper panel) and schematic diagram for DRE mutants (lower panel). Four Tax-response elements (TREs) and five potential AHR-response elements (DREs) are highlighted. (**B**) Dual luciferase reporter analysis in HEK293 cells co-transfected with HTLV-1 LTR-Luc of different length (-353/+402, -110/+402, -29/+402, +1/+402) (0.5 μg) and pcDNA3.1/pcDNA3.1-HA-AHR (0.5 μg). (**C, D**) Dual luciferase reporter analysis in HEK293 cells co-transfected with HTLV-1 LTR-Luc of various (**C**) DRE mutant (0.5 μg) or (**D**) DRE deletion (0.5 μg) and pcDNA3.1/pcDNA3.1-HA-AHR (0.5 μg). For each sample, 20 ng pRL-TK was used as an internal control. Results are shown as relative luciferase activity (firefly/renilla) normalized to that of the control group. (**E**) ChIP assay in HEK293 cells co-transfected with pGL4.22/HTLV-1 LTR-Luc (8 μg) and pcDNA3.1/pcDNA3.1-HA-AHR (8 μg). Precipitated DNA was detected by semiquantitative RT-PCR. Expression of transfection was analyzed by immunoblot. **, p<0.01. NS, not significant.

Using PROMO program (http://alggen.lsi.upc.es), we identified five putative DREs located on HTLV-1 LTR (Fig 4A). To determine whether these DREs are responsible for AHR-mediated transactivation of HTLV-1 LTR, various HTLV-1 LTR reporters of different length, DRE mutation (DREm) and DRE deletion (DREd) were constructed. We observed that AHR significantly induced the activity of wild-type LTR-Luc and LTR (-110/+402)-Luc, but not of LTR (-29/+402)-Luc and LTR (+1/+402)-Luc (Fig 4B), indicating the importance of DRE1 in regulating this response. Consistently, analysis of mutation/deletion of five DREs displayed that only mutation/deletion of DRE1 eliminated the AHR-induced reporter response (Fig 4C and 4D). Furthermore, we verified the binding of AHR to HTLV-1 LTR by chromatin immunoprecipitation (ChIP) assay (Fig 4E). All data above demonstrate that activated AHR drives HTLV-1 plus-strand transcription by direct binding to HTLV-1 LTR DRE1 site (CACGCATAT).

### Persistent NF-κB activation is critical for AHR overexpression in HTLV-1-infected T-cells

**Fig 5:**
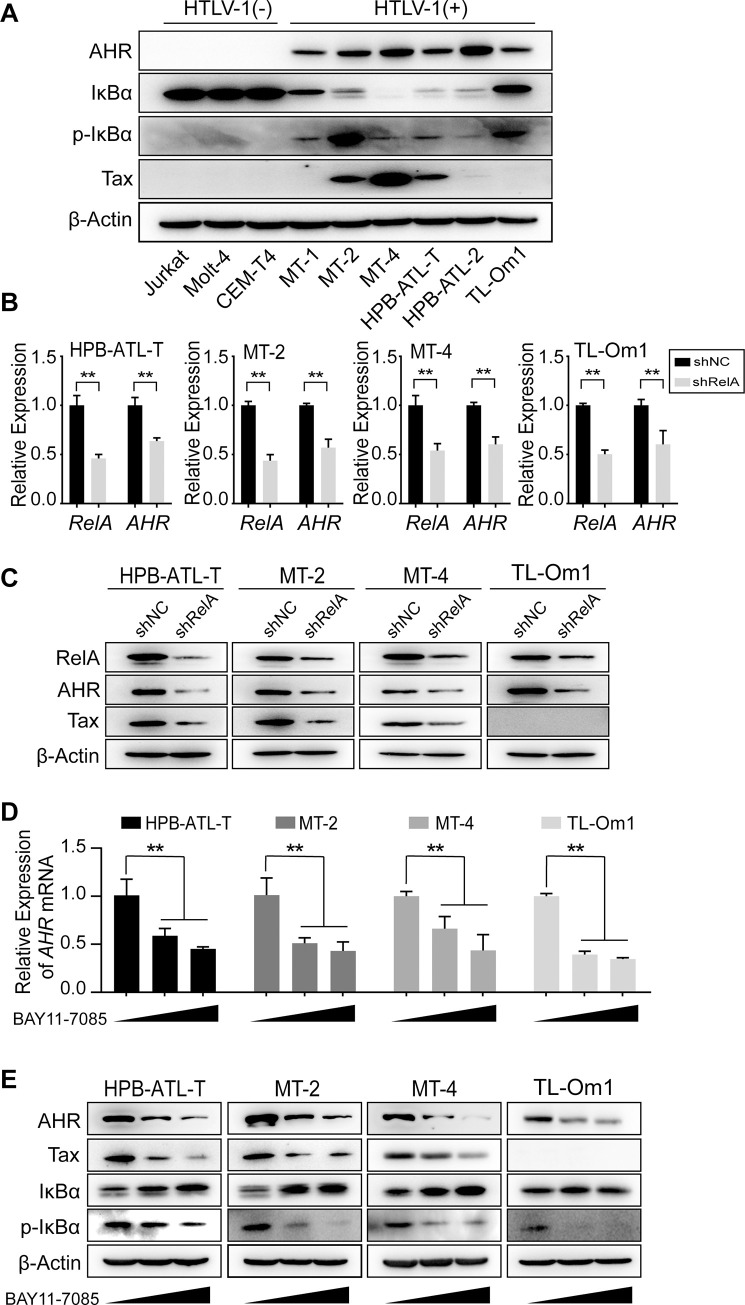
Persistent NF-κB activation is critical for AHR overexpression in HTLV-1-infected T-cells. (**A**) Immunoblot analysis of the level of AHR, Tax, IκBα and p-IκBα in each cell line. (**B, C**) HPB-ATL-T, MT-2, MT-4, TL-Om1 cells were infected with lentivirus against RelA. The screened RelA-knockdown cells were subjected to analyze the expression by (**B**) quantitative RT-PCR and (**C**) immunoblot. (**D, E**) HPB-ATL-T, MT-2, MT-4, TL-Om1 cells were treated with BAY11-7085 (2.5, 5 μM) or DMSO for 24 h, then subjected to analyze the expression by (**D**) quantitative RT-PCR and (**E**) immunoblot. Results of quantitative RT-PCR are shown as relative mRNA expression normalized to that of 18S rRNA. shNC, negative control of shRNA. p-IκBα, phosphorylated IκBα. *, p<0.05. **, p<0.01.

It is known that AHR is generally not/dimly expressed in lymphoid cell lines, whereas constitutive AHR overexpression was observed in HTLV-1-infected T-cell lines as well as primary ATL cells [16] (Fig 5A). What signals lead to AHR overexpression in these infected T-cells? Previously, Vogel et al. identified a response element for the nuclear factor kappa B (NF-κB) heterodimer RelA-p50 in AHR promoter, showing that lipopolysaccharide (LPS)-induced NF-κB activation resulted in AHR overexpression in a RelA-dependent manner [30]. Since persistent NF-κB activation was detected in most/all HTLV-1-infected T-cells [31, 32] (Fig 5A), we hypothesized that AHR overexpression in infected T-cells might be a consequence of the persistent NF-κB dysregulation. To test this hypothesis, we suppressed RelA expression in HPB-ATL-T, MT-2, MT-4 and TL-Om1 cells using lentivirus-mediated shRNA. As expected, knockdown of RelA greatly reduced AHR expression at both mRNA and protein levels (Fig 5B and 5C). Further supporting our hypothesis, treatment with BAY11-7085, an inhibitor of NF-κB activation and phosphorylation of NF-κB inhibitor alpha (IκBα), significantly impaired AHR expression in these four cell lines (Fig 5D and 5E). Given the important role of AHR signaling in HTLV-1 plus-strand expression, it is predictable that both knockdown of RelA and treatment with BAY11-7085 also attenuated Tax expression in HPB-ATL-T, MT-2 and MT-4 cells (Fig 5C and 5E). Collectively, these results indicate that persistent NF-κB activation is critical for AHR overexpression in HTLV-1-infected T-cells.

### The impact of Tax and HBZ on AHR

**Fig 6:**
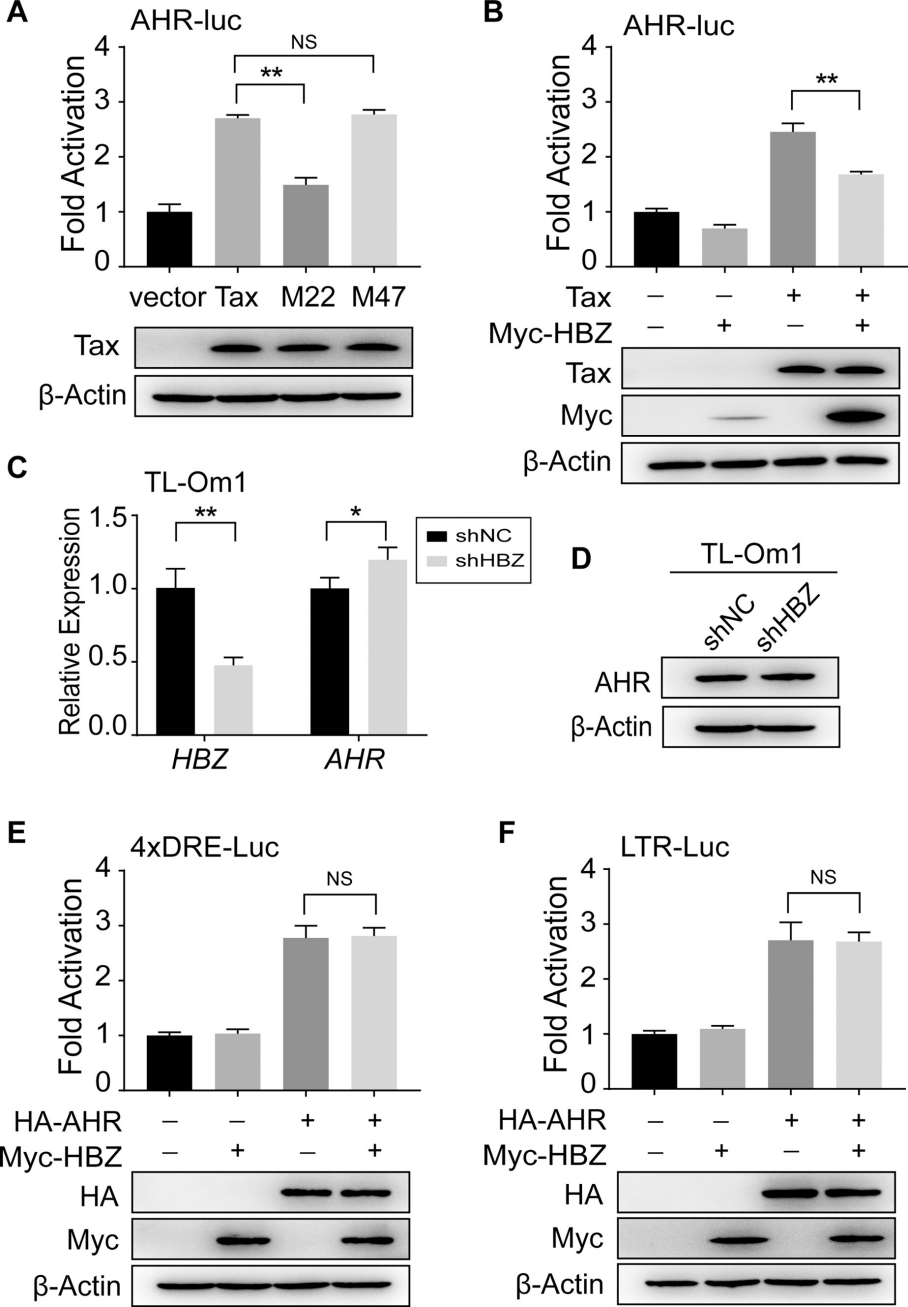
The impact of Tax and HBZ on AHR. (**A**) Dual luciferase reporter analysis in HEK293 cells co-transfected with AHR-luc (0.5 μg) and pCG/pCG-Tax/pCG-M22/pCG-M47 (0.5 μg). (**B**) Dual luciferase reporter analysis in HEK293 cells co-transfected with AHR-Luc (0.5 μg) and pCG/pCG-Tax (0.5 μg) and pcDNA3.1/pcDNA3.1-Myc-His-HBZ (0.5 μg). Expression of HBZ was greatly elevated after co-transfection with Tax, which might due to that Tax can potently increase the expression level of pcDNA3.1 vector. (**C, D**) TL-Om1 cells were infected with lentivirus against HBZ. The screened HBZ-knockdown cells were subjected to analyze the expression by (**C**) quantitative RT-PCR and (**D**) immunoblot. (**E**, **F**) Dual luciferase reporter analysis in HEK293 cells co-transfected with (**E**) 4xDRE-luc (0.5 μg) or (**F**) HTLV-1 LTR-luc (0.5 μg) and pcDNA3.1/pcDNA3.1-AHR (0.5 μg) and pcDNA3.1/pcDNA3.1-Myc-His-HBZ (0.5 μg). For each sample, 20 ng pRL-TK was used as an internal control. Results are shown as relative luciferase activity (firefly/renilla) normalized to that of the control group. Expression of transfection was analyzed by immunoblot. shNC, negative control of shRNA. *, p<0.05. **, p<0.01. NS, not significant.

Tax and HBZ counteract each other in many signaling pathways, including canonical NF-κB, CREB, activator protein 1 (AP-1) and transforming growth factor beta (TGF-β). Molecular study has revealed that Tax activates both canonical and noncanonical NF-κB pathways through direct and indirect interactions with NF-κB essential modulator (NEMO) and IκB kinase (IKK) complex [33–38]. On the contrary, HBZ selectively inhibits canonical NF-κB activation by impairing RelA DNA binding ability and promoting RelA degradation through ubiquitination-dependent pathway [39]. In the previous study of Hayashibara et al., by using a CdCl2-inducible Tax-expressing cell line JPX-9, they had reported that increased Tax expression could elevate AHR expression [16]. To further investigate how Tax elevates AHR expression, we analyzed two well-characterized Tax mutants, M22 (Tax^T130A, L131S^, defective in NF-κB activation) and M47 (Tax^L319R, L320S^, unable to activate the CREB/ATF pathway) [40]. We found that M22, rather than M47, lost the ability to activate AHR-Luc (Fig 6A), indicating that Tax elevates AHR expression via activating NF-κB. Thus, Tax would contribute to AHR expression in Tax-expressing cells by activating NF-κB; conversely, NF-κB-AHR axis might contribute to Tax-mediated transactivation of HTLV-1 LTR when AHR ligands are sufficient, which is compatible with previous observation that the capability of M22 to activate HTLV-1 LTR-luc was only ~50% of wild-type Tax in Jurkat cells [40].

Since HBZ suppresses canonical NF-κB activation, it is unsurprising that Tax-induced activity of AHR-Luc decreased after co-transfection with HBZ (Fig 6B). It should be noticed that NF-κB activation is persistent not only in Tax-expressing cells but also in those without Tax expression, e.g. TL-Om1 (Fig 5A), a possible consequence of many gain-of-function and loss-of-function mutations in T cell receptor (TCR)–NF-κB pathway [41]. Then, we further investigated the impact of HBZ on AHR expression in TL-Om1 cells. In accord with the result of reporter assay, knockdown of HBZ slightly increased *AHR* mRNA in TL-Om1 cells (Fig 6C), although there was no apparent increase in AHR protein (Fig 6D). Considering that HBZ has been reported to heterodimerize with various transcription factors—such as CREB, c-Jun, JunB and ATF-3—and hinder their transcriptional capability [42, 43], we examined whether HBZ affects AHR-mediated transactivation. We found that HBZ did not influence AHR-mediated activation of 4xDRE-Luc and HTLV-1 LTR-Luc (Fig 6E and 6F).

## Discussion

Many evidences indicate that HTLV-1 is not completely silent *in vivo*. However, little is known about where and how HTLV-1 achieves its reactivation. In this study, we utilized MT-1 cells, in which the pattern of viral gene expression is thought equivalent to that of primary HTLV-1-infected T-cells (when cultured in basic cell culture medium), to show that HTLV-1 latency-reactivation-latency switching could be manipulated by adding and removing additional AHR ligands (e.g. kynurenine) (Fig 2). This result strongly supports a model that the pattern of HTLV-1 gene expression is variable in response to the level of AHR ligands in the milieu. Moreover, we explicate the underlying mechanism: activated AHR binds to HTLV-1 LTR DRE site (CACGCATAT) and drives plus-strand transcription (Fig 4). Since a major obstacle for HTLV-1 to establish robust plus-strand expression is HBZ-mediated transcriptional suppression, it pushes us to further investigate the interplay between AHR and HBZ. We found that AHR signaling had no direct impact on *HBZ* transcription (Fig 1E and 1F). On the other hand, although HBZ would constrict AHR expression (Fig 6B and 6C) (the fact is that AHR is still overexpressed in HTLV-1-infected T-cells), it did not affect AHR-mediated transactivation of viral LTR (Fig 6F). Therefore, AHR is an ideal candidate for HTLV-1 to overwhelm HBZ-mediated transcriptional suppression and reconstruct plus-strand expression. Taken together, our findings imply that AHR, an environmental sensor, might be employed by HTLV-1 to accomplish latency-reactivation switching *in vivo*.

### The cues for HTLV-1 reactivation: AHR ligands

Background AHR signaling is ongoing in HTLV-1-infected T-cell lines due to the existence of endogenous ligands (e.g. tryptophan metabolites) in cell culture medium, and we show that this background AHR signaling was inextricably linked to plus-strand expression in HPB-ATL-T, MT-2 and MT-4 cells (Fig 1). Thus, when assessing HTLV-1 reactivation *in vitro*, the background AHR signaling should be taken into account. It is assumed that AHR ligands in peripheral blood are ordinarily kept at a relatively stable level due to the excretory system, whereas in cell culture dish the ligands should accumulate with incubation, contributing to background AHR signaling. The different ligand dynamics *in vivo* versus *in vitro* might provide a reasonable explanation for a strange phenomenon—the spontaneous transcriptional activation of HTLV-1 in freshly isolated PBMCs of infected individuals, as one common characteristic of this activation is that the isolated PBMCs generally require incubation *in vitro* for hours [44].

Alteration of systemic tryptophan metabolism has been reported to be implicated in HTLV-1 pathogenesis. Masaki et al. showed that the ratio of serum kynurenine/tryptophan and the concentration of kynurenine in HTLV-1 asymptomatic carriers (ACs) were significantly higher than those in healthy controls. Both increased significantly with the progression from HTLV-1 AC to ATL and acted as significantly independent detrimental prognostic factors in ATL [45]. As kynurenine-activated AHR was capable to reactivate the latent virus in MT-1 cells in a ligand dose-dependent manner (Fig 2), our findings reveal a molecular mechanism for the observed association between the disordered tryptophan metabolism and HTLV-1 pathogenesis. Notably, in addition to endogenous ligands, heterogeneous exogenous ligands are also known to activate AHR, e.g. flavonoids and indoles from diet and benzo[a]pyrene from smoking [46]. Whether accumulation of systemic ligands absorbed from diet and smoking contributes to HTLV-1 pathogenesis will be another topic needed to be investigated.

Interestingly, Hayashibara et al. reported that 9/10 samples of primary ATL cells (PBMCs) exhibited AHR overexpression while apparent AHR signaling was only detected in 1/10 sample (by measuring *CYP1A1* mRNA) [16], suggesting that the level of systemic ligands in peripheral blood is usually too low to support efficient AHR signaling. This observation to some extent is in accord with the rare detection of viral products in freshly isolated PBMCs of infected individuals. We therefore speculate that HTLV-1 reactivation may not/seldom occur in peripheral blood but rather in certain anatomical compartments that are prone to locally accumulate ligands, such as bone marrow (or other lymphoid organs/tissues). A supporting evidence is that Tax is highly expressed in bone marrow of Japanese macaques infected with Simian T-lymphotropic virus type 1 (STLV-1, the simian counterpart of HTLV-1) [47]. Further investigations are still required in the future to clarify the following points: (i) the exact ligand types and their level in these compartments; (ii) the threshold of each ligand type to reactivate HTLV-1 from latency; (iii) it is obvious that the pattern of HTLV-1 gene expression in MT-1 cells is quite distinct from those in HPB-ATL-T, MT-2 and MT-4 cells under the background AHR signaling (when cultured in basic cell culture medium). What contributes to these differences? (iv) why the induced Tax could not sustain HTLV-1 reactivation in MT-1 cells after removal of the additional AHR ligands (Fig 2)?

### Crosstalk between AHR, Tax and NF-κB

A fundamental event in HTLV-1 pathogenesis is the persistent NF-κB dysregulation, which is critical for the proliferation and survival of infected T-cells [38]. In this study, we found that this persistent NF-κB activation constitutes one key prerequisite for AHR overexpression in infected T-cells. Indeed, inactivation of NF-κB significantly reduced AHR expression in HPB-ATL-T, MT-2, MT-4 and TL-Om1 cells (Fig 5). Intriguingly, Tax can elevate AHR expression via activating NF-κB (Fig 6A). The AHR-Tax circuit might partly answer why AHR signaling is effective in inducing HTLV-1 plus-strand expression. Thus, it is not surprising that plus-strand expression (we used Tax expression as a hallmark) was also impaired after NF-κB inactivation (Fig 5C, E). In addition, AHR has been reported to control the transcriptional programs of NF-κB by binding to NF-κB members (RelA and RelB) [26, 27], that is, there might exist additional AHR positive feedback loop in infected T-cells, which adds a layer of complexity to the interaction between AHR, Tax and NF-κB.

Various stimuli—such as extracellular antigens, inflammation cytokines, cytoplasmic oxidative stress and nuclear DNA damage—are known to activate NF-κB. Therefore, potential linkage between these factors and HTLV-1 plus-strand expression is established (when AHR ligands are sufficient). It has been reported that several stress-inducing agents [12-O-tetradecanoylphorbol-13-acetate (TPA), cisplatin, etoposide, taxol, 3-methylcholanthrene (3-MC, a known AHR ligand) and H_2_O_2_] triggered HTLV-1 plus-strand expression [24, 48], but the underlying mechanisms are still not well explicated. Since cytotoxic stresses drive both NF-κB and AHR signaling [49, 50], we speculate that such agents induce plus-strand expression possibly through NF-κB-AHR axis.

In summary, we here reveal a role of AHR in HTLV-1 plus-strand expression and its capability to reactivate HTLV-1 from latency. Our findings provide a novel insight into where and how HTLV-1 might achieve its reactivation *in vivo* and address AHR as a potential target for prophylaxis and treatment of HTLV-1-related diseases.

## Materials and methods

### Cell culture

MT-1, HPB-ATL-T, HPB-ATL-2, TL-Om1 are ATL-derived T-cell lines. MT-2 and MT-4 are HTLV-1-transformed T-cell lines. Jurkat, Molt-4 and CEM-T4 are HTLV-1-negative T-cell lines. All these T-cell lines were obtained from Prof. Masao Matsuoka (Kumamoto University, Japan) and cultured in RPMI1640 (Gibco) supplemented with 10% FBS (Gibco). HEK293 was purchased from Shanghai Institute of Cell Biology and cultured in DMEM (Gibco) supplemented with 10% FBS.

### Reagents and antibodies

CH-223191 was purchased from Selleck. L-kynurenine and BAY11-7085 were purchased from MedChemExpress. Antibodies were used as following: AHR (D5S6H; Cell Signaling Technology), RelA (D14E12; Cell Signaling Technology), IκBα (L35A5; Cell Signaling Technology), phospho-IκBα (14D4; Cell Signaling Technology), α-Tubulin (DM1A; Cell Signaling Technology), LaminB1 (D9V6H; Cell Signaling Technology), HTLV-1 Tax (1A3; Abcam), HTLV-1 gp46 (67/5.5.13.1; Abcam), HTLV-1 p24 (46/3.24.4; Abcam), HTLV-1 p19 (TP-7; Abcam), β-Actin (AF0003; Beyotime Biotechnology), GAPDH (AF0006; Beyotime Biotechnology).

### Plasmids

pcDNA3.1-HA-AHR was constructed by cloning human AHR cDNA with HA tag into pcDNA3.1(-) vector (Invitrogen). pCG-Tax and pcDNA3.1-Myc-His-HBZ were described previously [51]. 4xDRE-Luc was constructed by cloning four copies of DRE within human CYP1A1 promoter in front of a TATA box into pGL4.22 vector (Promega) [25]. AHR-Luc and HTLV-1 LTR-Luc were constructed by cloning human AHR promoter (nt -2000 to +200, relative to the transcription start site) and HTLV-1 LTR (GenBank: J02029.1, nt +23 to +777) into pGL4.22 vector, respectively. LTR (-110/+402), LTR (-29/+402) and LTR (+1/+402) were constructed by cloning HTLV-1 LTR of different length into pGL4.22 vector. DRE1m, DRE2m, DRE3m, DRE4m, DRE5m, DRE1d, DRE2d, DRE3d, DRE4d, DRE5d were generated from HTLV-1 LTR-Luc. The detailed information of HTLV-1 LTR mutants is shown in Fig 4A. pcDNA3.1-HA-AHR^A78D^ were generated from pcDNA3.1-HA-AHR. pCG-M22 (Tax^T130A, L131S^) and pCG-M47 (Tax^L319R, L320S^) were generated from pCG-Tax. For site mutant, we used Mut Express II Fast Mutagenesis Kit V2 (Vazyme).

### RNA extraction and quantitative RT-PCR

Total RNA extraction was performed with Trizol (Invitrogen) according to the manufacturer’s instructions. cDNA was synthesized using HiScript II Q RT SuperMix (Vazyme). Quantitative RT-PCR was performed using AceQ qPCR SYBR Green Master Mix (Vazyme).

The following primers were used:

18S rRNA forward 5’-GTTCTTAGTTGGTGGAGCGATTTG-3’;

18S rRNA reverse 5’-TTGCTCAATCTCGGGTGGC-3’;

Human *AHR* forward 5’-GCCAACATCACCTACGCCAGTC-3’;

Human *AHR* reverse 5’-TCGGTCTCTATGCCGCTTGGAA-3’;

Human *RelA* forward 5’-ATGTGGAGATCATTGAGCAGC-3’;

Human *RelA* reverse 5’-CCTGGTCCTGTGTAGCCATT -3’;

HTLV-1 *gag* forward 5’-AGCCCCCAGTTCATGCAGACC-3’;

HTLV-1 *gag* reverse 5’-GAGGGAGGAGCAAAGGTACTG-3’;

HTLV-1 *env* forward 5’-CGTCCGCCGTCTAGCTTCC-3’;

HTLV-1 *env* reverse 5’-ATTGTGAGAGTACAGCAGC-3’;

HTLV-1 *tax* forward 5’-ACCAACACCATGGCCCA-3’;

HTLV-1 *tax* reverse 5’-GAGTCGAGGGATAAGGAAC-3’;

HTLV-1 *HBZ* forward 5’-ATGGCGGCCTCAGGGCT-3’;

HTLV-1 *HBZ* reverse 5’-CTTCTAAGGATAGCAAACCGTCAAG-3’.

### Immunoblot

Cells were lysed in radioimmunoprecipitation assay (RIPA) buffer (Beyotime Biotechnology) supplemented with protease inhibitor cocktail (Thermo Scientific) at 4°C for 30 min. Lysates were cleared by centrifugation at 13,000 g for 10 min at 4°C. The extracts were subjected to SDS-polyacrylamide gel electrophoresis and further transferred onto nitrocellulose membrane (Thermo Scientific). The membrane was blocked in milk at 4°C overnight then probed with diluted specific antibody for 3 h at room temperature. After washing with 5% Tween–phosphate-buffered saline (PBS) for 10 min (3 times), the membrane was incubated in appropriate horseradish peroxidase (HRP)-conjugated secondary antibodies for 1 h at room temperature. After that, the membrane was washed with 5% Tween-PBS for 10 min (3 times), and the protein band was visualized with BeyoECL Star (Beyotime Biotechnology).

For nuclear and cytosolic fraction separation, we used Nuclear and Cytoplasmic Protein Extraction Kit (Beyotime Biotechnology) according to the manufacturer’s instructions.

### Lentivirus transduction

Lentiviruses were purchased from Genechem.

The shRNA sequences were:

5’-TTCTTTGATGTTGCATTAA-3’ targeting human *AHR*;

5’-GATTGAGGAGAAACGTAAA-3’ targeting human *RelA*;

5’-ACAGCATAGTGCTAGGAAA-3’ targeting HTLV-1 *HBZ*;

5’-TTCTCCGAACGTGTCACGT-3’ as negative control.

The map of the lentivirus backbone vector GV493 is shown at http://www.genechem.com.cn/index/supports/zaiti_info.html?id=83. Lentivirus transduction was performed by supplementation of 4 μg/ml polybrene. Transduced cells were selected with 0.5 μg/ml puromycin.

### Cell transfection

Cell transfection was conducted using Lipofectamine 3000 Transfection Reagent (Invitrogen) according to the manufacturer’s instructions. In brief, HEK293 cells were seeded to be 70–90% confluent at transfection. For 12-well plate/10 cm dish transfection, 1–1.5 μg/16 μg plasmids were used. The ratio of plasmids: p3000 reagent: Lipofectamine 3000 reagent is 1 μg: 2 μL: 3 μL.

### Luciferase assay

HEK293 cells were seeded on 12-well plate and transfected with the indicated plasmids when cells are 70–90% confluent. For each well, 20 ng renilla luciferase reporter plasmid pRL-TK (Promega) was used as an internal control. 48 h after transfection, luciferase activity was measured with Dual-Luciferase Reporter Assay System (Promega) according to the manufacturer’s instructions.

### Chromatin immunoprecipitation (ChIP)

ChIP assay was performed using Pierce Magnetic ChIP Kit (Thermo Scientific) according to the manufacturer’s instructions. In brief, HEK293 cells were seeded on 10 cm dish and transfected with the indicated plasmids when cells are 70–90% confluent. 48 h after transfection, 8 × 10^6^ cells (for 2 ChIP, the remaining cells were subjected to analyze expression by immunoblot) were cross-linked by 1% formaldehyde (Sigma) for 10 min, and then quenched using glycine for 5 min. Cross-linked chromatin was digested into size around 150–900 bp using micrococcal nuclease. 10/200 μL sheared chromatin was used as common 10% input. ChIP (90 μL sheared chromatin for each) was performed with 2 μg HA antibody or equal normal rabbit IgG at 4°C overnight with rotation. Precipitated DNA was detected by semiquantitative RT-PCR under the following conditions: 3 minutes at 95°C for denaturation, 25 cycles of 15 sec at 95°C, 15 sec at 63°C, 10 sec at 72°C and 5 min at 72°C for final extension.

The specific primers for HTLV-1 LTR containing DRE1:

forward 5’-CAGGCGTTGACGACAACCC-3’;

reverse 5’-GTGGATGGCGGCCTCAGGTA-3’.

### Statistical analysis

Statistical significance was assessed by Student’s *t*-test or analysis of variance (ANOVA) using GraphPad Prism 5 (Graphpad software).

## Supporting information

S1 Fig CH-223191 inhibits AHR nuclear translocation in HTLV-1-infected T-cells. HPB-ATL-T, MT-2, MT-4 and TL-Om1 cells were treated with CH-223191 (10, 20 μM) or DMSO for 24 h, then subjected to analyze the level of cytosolic and nuclear AHR by immunoblot. α-Tubulin and LaminB1 were used as control for cytosolic and nuclear fraction respectively.
(TIF)
