# Supplementary figures and images for: AHR is a tunable knob that controls HTLV-1 latency-reactivation switching
Source: PLoS Pathog. 2020 Jul 17;16(7):e1008664. doi: 10.1371/journal.ppat.1008664 (PMC7367443; doi:10.1371/journal.ppat.1008664)

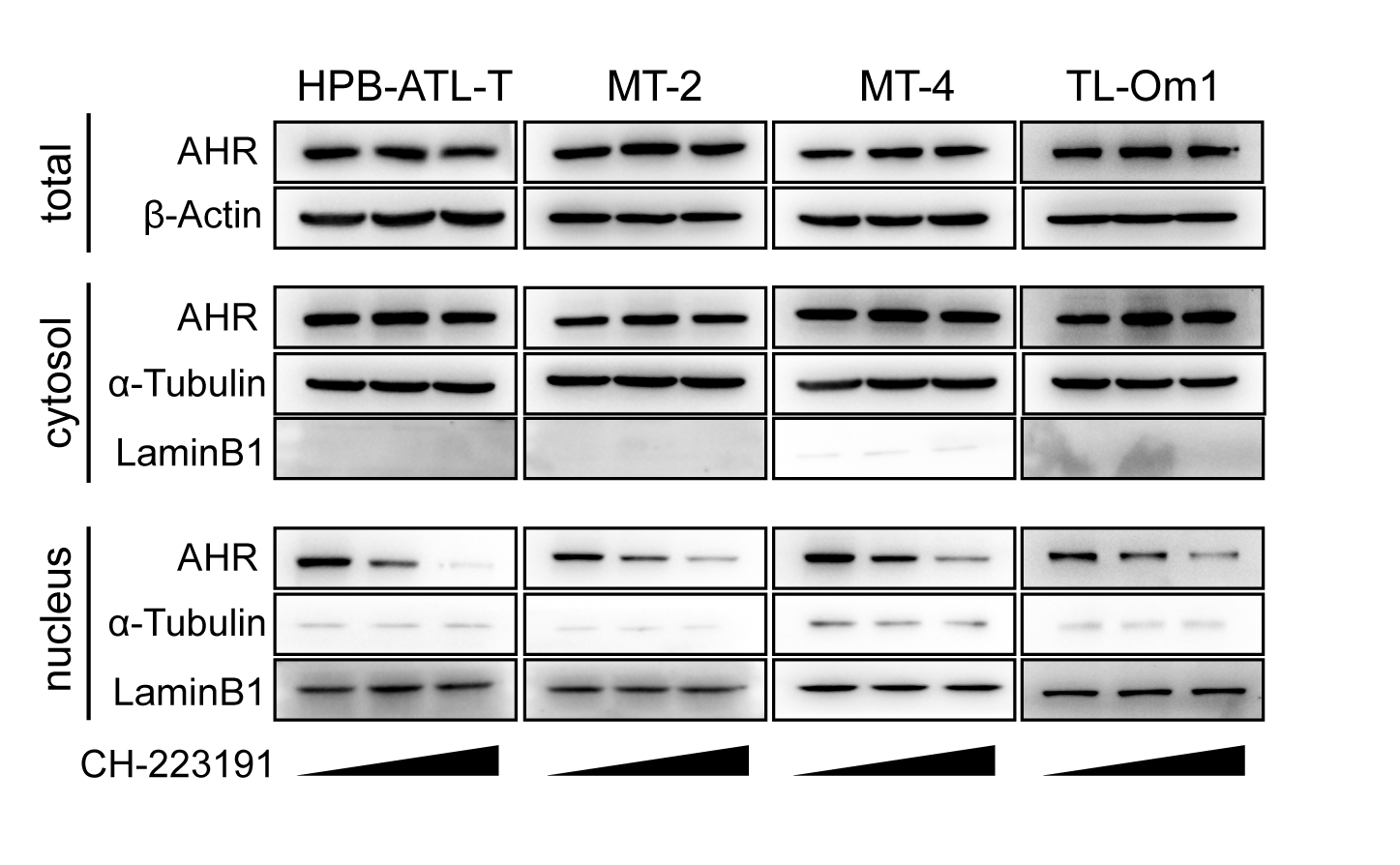

Supplement: S1 Fig — HPB-ATL-T, MT-2, MT-4 and TL-Om1 cells were treated with CH-223191 (10, 20 μM) or DMSO for 24 h, then subjected to analyze the level of cytosolic and nuclear AHR by immunoblot. α-Tubulin and LaminB1 were used as control for cytosolic and nuclear fraction respectively. (TIF) [file ppat.1008664.s001.tif]
